# Physical and social wellbeing of family caregivers of persons with hepatitis B associated chronic liver disease in Ghana: a qualitative study

**DOI:** 10.1186/s12875-023-02041-5

**Published:** 2023-03-24

**Authors:** Dora Abaah, Lillian Akorfa Ohene, Charles Ampong Adjei

**Affiliations:** University of Ghana, Accra, Ghana

**Keywords:** Hepatitis B, Family caregivers, Wellbeing, Ghana

## Abstract

**Background:**

Hepatitis B is one of the most common viral (HBV)infections that affect the liver. Infection with the virus may result in varying severity of liver disease which may be acute or chronic. Though most people recover from the infection, about 5 − 10% of cases lead to chronic infection. Persons who develop HBV-related debilitating liver disease will likely require informal care from family caregivers.

**Aim:**

This study sought to explore the physical and social wellbeing of family caregivers of persons with hepatitis B-associated chronic liver disease in a tertiary hospital in the Central region of Ghana.

**Methods:**

This study adopted an exploratory, descriptive qualitative research design. We used a purposive sampling technique and a semi-structured interview guide to interview eighteen participants. The Quality of Life (QoL) model applied to family caregivers underpinned the study and guided the formulation of study objectives. Data analysis followed Braun and Clarke’s procedure for thematic content analysis. Similar codes were grouped into subthemes, and similar subthemes were grouped into major themes. The consolidated criteria for reporting qualitative research (coreq) checklist was used as a guide for writing the study.

**Results:**

Two major themes emerged from the study: physical wellbeing and social wellbeing. Seven subthemes were also identified: physical body changes and physiological body changes (physical wellbeing) and role strain, social isolation, financial impact, affection/sexual function, and support social wellbeing). These central themes aligned with two domains of the QoL model applied to family caregivers.

**Conclusion:**

Family caregivers of persons with HB-associated liver disease suffer ill health due to the burden of physical care for their sick relatives and neglect their health due to time constraints. They also experience role strain as they cannot attend to other family responsibilities and feel socially isolated since they spend all their time caring for a sick family member.

## Background

The Hepatitis B virus (HBV) is one of the most common liver infections globally [1]. An estimated 2 billion people are infected with HBV, and about 296 million live with chronic hepatitis B infection in 2019 [2, 3]. Most adults infected with the virus recuperate, but 5-10% cannot clear the virus and become chronically infected. Most people with chronic infections have a mild liver disease with little or no long-term illness or death [4]. In 2019, HBV-related liver diseases resulted in an estimated 820,000 deaths worldwide [5]. Most of these deaths are caused by cirrhosis and hepatocellular cancer [6].

Around 4% of the world’s population lives in areas where HBV infection is endemic [2]. HBV infection is highest in the WHO Western Pacific Region and the WHO African Region, where 116 million and 81 million people are chronically infected [2]. In Africa, about 8% of the total population has chronic HBV infection, which puts them at greater risk for chronic liver disease; also, 30-50% of cirrhosis deaths result from chronic HBV infection [7, 8]. In Ghana, a systematic review by Abesig et al. [9] revealed a prevalence of HBV, as measured by HBsAg seropositivity, to be 8.36%. A study by Blankson et al. [10] revealed that an estimated 40% of patients reporting cirrhosis have HBV infection. The Central Region of Ghana has a hepatitis B prevalence estimate of 11.5% [11]. The fact that some HBV infections progress to HB-related chronic liver diseases suggests that patients are likely to require some form of informal care from family members. A family caregiver is a person who provides physical, mental, and financial support to a family member who is unable to care for themselves due to disease, accident, or disability [12]. Evidence indicates that illness affects the sick individual and the family [13].

Sherman [14] has reported that the family caregiver’s needs are often overlooked, with the focus mainly on the patient. Caregiving for a person with severe chronic illness generally entails physically demanding activity over a long period. Caregivers are more likely to neglect their health, which can result in musculoskeletal injuries, arthritis aggravation, and other chronic conditions. As the sick person’s symptom burden increases, so do the caregiver’s physical requirements [15]. Patients’ health and the health of their family caregivers have been proven to be tightly linked, implying that supporting the caregiver’s health will benefit the patient’s health and vice versa. Throughout sickness, the influence of caregiving on the social wellbeing of family caregivers is essential. A family member diagnosed with HB-related chronic liver disease will most likely require informal care from family members. The demanding and prolonged nature of informal care for a chronic illness may affect the family’s social structure [16]. Social support is, therefore, necessary to ensure caregiver comfort since studies have indicated that a lack of support from other family members significantly influences the health of family caregivers. Even though family caregivers frequently suffer social isolation and withdrawal, those receiving the most social assistance have the lowest depression and caregiver stress. The high prevalence of hepatitis B infection in the Central Region suggests increased chronic cases. Some of these chronic cases are likely to progress to chronic liver diseases. Hence the need for support from family caregivers. Therefore, this study aimed to explore the physical and social wellbeing of family caregivers of persons with hepatitis B- associated chronic liver disease.

## Methods

### Study aim and design

The study aimed to explore the physical and social wellbeing of family caregivers of persons with hepatitis B-associated chronic liver disease. The study adopted an exploratory, descriptive qualitative design. This design enabled participants to share experiences about their physical and social wellbeing in caring for a family member with HBV-related chronic liver disease [17]. The theoretical framework of Quality of Life Model Applied to the Family Caregivers model was used to explore the wellbeing of family caregivers of persons with HB-associated chronic liver disease. The model’s constructs helped explore the physical, social, psychological, and spiritual domains of the wellbeing of the family caregivers.

### Research setting

Participants were recruited from the Cape Coast Teaching Hospital (CCTH) in the Central region of Ghana. It is a public facility and serves as the main referral hospital in the region. Persons diagnosed with HBV infection attended the hepatitis clinic on special clinic days in the Outpatient Department. The clinic has an average attendant of twenty patients. Patients who were critically ill from HBV-related chronic liver disease were on admission to the Male or Female Medical Wards for further management [18]. Persons who were not on admission but looked critically ill were accompanied to the hepatitis B clinic by their family caregivers. The CCTH Ethics Review Committee approved ethics on 26th October 2020 (Protocol No. CCTHERC/EC/2020/091). The unit in-charges permitted access to the unit for data collection to commence.

### Participants and recruitment

The participants in this study were family caregivers who lived in the Cape Coast Metropolis and provided care for a relative diagnosed with HB-related chronic liver disease. The sick persons received treatment at the Cape Coast Teaching Hospital. Also, participants had to be at least 18 years of age. The study excluded employed informal caregivers who were not family related. Family caregivers with difficulty in communication were also excluded from the study. A total of 18 family caregivers, determined by data saturation, participated in the study [19, 20]. We used purposive sampling to recruit participants who met the inclusion criteria by contacting caregivers who provided informal care for their relatives diagnosed with HB-associated chronic disease and had accompanied the patients to the hospital. Eleven caregivers were providing care for family members with HB-related liver cirrhosis, two had patients with hepatic encephalopathy, and one had a patient with hepatocellular carcinoma. The remaining four were newly diagnosed and required frequent monitoring and treatment. Fourteen participants had relatives currently on admission to the ward, and four had accompanied their relatives to the OPD clinic for review. Data collection lasted from October 2020 to February 2021. We visited the Outpatient Department and the Male and Female Medical Wards on clinic days to meet and inform family caregivers about the study.

The purpose of the study was explained to each participant, after which each signed a consent form to indicate their willingness to participate. Interviews then started, and probing was used to gather relevant information. The interview venue was outside the Male or Female Medical wards and participants’ homes. Depending on their convenience, interested participants were contacted by phone to arrange a meeting day and venue. The participants selected the venues based on convenience. Fourteen participants who had patients on admission preferred venues in the hospital, whereas the remaining four who did not have patients on admission chose their homes.

Interviews lasted between 45 and 60 min. The interviewer used an audio recorder to record the discussions and took short notes while interviewing participants. The first author recorded participants’ nonverbal communication as she interviewed them. She also noted the mannerisms of the participants and anything of relevance in a field diary to help the researcher better understand the information gathered. Data reached saturation after the 15th interview[19]. Nonetheless, three more interviews were conducted to ensure no new information emerged.

### Research instrument

The research team developed a semi-structured interview guide to conduct face-to-face interviews with each participant for this study. Two primary domains of the QoL model applied to family caregivers by Ferrell (2001) informed the central questions in the interview guide. The sub-themes in the model formed the probes for the specific questions. The interview guide was piloted with two family caregivers caring for their relatives on admission for treatment at the CCTH before it was used for the main participants [17]. This was done to ensure that the questions elicited the appropriate responses. However, the reactions from the pilot test were not included in the main study. Participants were mainly asked to share their experiences on their physical and social wellbeing since they took up caregiving of a relative suffering from HB-related chronic liver disease. All interviews were conducted by the first author.

### Data analysis

Thematic content analysis was done manually following Braun and Clarke’s procedure for thematic content analysis [21]. The thematic content analysis enabled the researchers to analyze data based on the QoL framework. After each interview, the researchers listened to the audio recordings and transcribed the interviews verbatim. We read through the transcripts multiple times to familiarize ourselves with the data. The authors discussed the unique codes and categorized them to generate themes. The authors discussed different codes and categories to reach a consensus on the identified themes. Themes and subthemes were then defined and named.

## Results

### Demographic characteristics

This study involved 18 participants aged between 19 and 65 years. Other demographic characteristics are presented in Table 1. The participants were all Ghanaians living in the Cape Coast Metropolis. All participants were married with children except for one participant who was single with no child. Among the 18 participants, 6 were spouses, 7 were parents, 4 were siblings, and 1 was a nephew. All participants had rendered care between 4 months to 2 years. Thirteen participants were self-employed as businessmen, artisans, farmers, beauticians, and petty traders. Two participants were public servants, two were in private practice, and one was an apprentice.

**Table 1:** Demographic Characteristics of participants

Participant (ID)	Age (Years)	Occupation	Duration of caregiving (Yrs)	Relationship
P1	42	Teacher	2years	Spouse
P2	48	Petty Trader	1year	Parent
P3	35	Teacher	6months	Spouse
P4	51	Trader	1year	Spouse
P5	35	Farmer	1 year	Sibling
P6	36	Petty trader	2year**s**	Parent
P7	43	Trader	1 year	Parent
P8	29	Artist	6 months	Nephew
P9	63	Farmer	2 years	Spouse
P10	37	Mason	1 1/2 years	Spouse
P11	65	Farmer	4 months	Parent
P12	33	Cleaner	6 months	Parent
P13	23	Hairdresser	1 year	Parent
P14	36	Trader	6months	Parent
P15	37	Mechanic	1 year	Sibling
P16	32	Statistician	6months	Sibling
P17	19	Apprentice	4months	Sibling
P18	55	Businessman	1year	Spouse

### Themes and subthemes

Table 2 presented the two major themes and six sub-themes identified from this study. The major theme, physical wellbeing had its sub-themes identified as, physical body changes and physiological body changes. Under the major theme of social wellbeing, subthemes were identified as role strain, social isolation, financial impact and support and affection/sexual function.

**Table 2:** Summary of Themes and Subthemes

THEMES	SUBTHEMES
PHYSICAL WELLBEING	Physical body changes Physiological changes
SOCIAL WELLBEING	Role strain Social isolation Financial impact and support Affection/Sexual function

### The physical wellbeing of family caregivers

According to the participants’ narratives, the demands and burden of caring for adult sick persons are complex and physically exhausting. Additionally, the chronicity of hepatitis B-associated liver disease prolongs care, with related consequences on the caregivers’ physiological functions. *The findings under physical wellbeing were concerns the caregivers raised about the body changes since they assumed the caregiver roles.*

### Physical body changes

The participants reported several physical bodies changes such as weight loss, poor personal grooming, and change in their physique. For example, most participants reported weight loss because of their engagement in physical caregiving activities, including carrying and lifting the sick and helping the ill achieve primary care needs such as bathing and feeding. The caregivers also became errands persons for the sick, and in that regard, they ensured that the sick met all hospital and other essential care commitments. According to the participants, meeting the physical needs of ill persons mainly was to the detriment of their wellbeing.

*“I support him to go everywhere. For these two months, I hardly slept for more than 2 hours because of his frequent visits to the toilet. I have lost weight because of that” (P*4).

*“Taking a car in and out all the time to the hospital is a very tiring task for me. Because of the movement up and down, I sometimes forget to eat, and sometimes too by the time I get home it is late, so I can’t eat. I have reduced in weight “(P*5).

The participants also raised concerns about their unmet physical needs. For example, many of them felt that the health needs of their sick relatives outweigh their need to attend to their grooming. Therefore, only some caregivers can visit hair salons, barbershops, or beauty spas.

*“Ei madam there is nothing like physical wellbeing, especially anytime he gets seriously sick. Look at my hair, I have been wearing this wig cap for a long time. No time to even visit the salon” (P*1)

*“I wish to visit the barber and look at my hair. I spend all my time here at the hospital” (P*16).

Taking up a caregiver role was a sacrifice and a compromise of meeting personal health needs. Some participants stopped visiting the gym, doing early morning walks, or jogging to make time to care for the sick. These participants reported that they could see noticeable changes in their bodies due to the lack of exercise.

*“I used to visit the gym for training, but now I cannot do so because of a lack of time since I have to be with my uncle most of the time. I can see that I am losing form; my body shape has changed” (*P8).

*“My inability to go for my usual early morning walk is making my body posture change”* (P18).

### Physiological changes

The participants reported physiological changes as a shared experience in caregiving. These changes included loss of appetite, nausea, constipation, fatigue, inadequate sleep, and body aches and pains. The loss of appetite was mainly associated with the severity of the patient’s condition, increased care demands, and hospitalization. For example, some participants reported that the environment and smells from the hospital caused them to lose their appetite. According to them, unpleasant sights and odors in the hospital caused nausea. Some also indicated that washing dirty linen and cleaning up their sick relatives gave them vomiting symptoms.

*“Madam, as for the appetite, it’s not there. I am unable to eat in the hospital environment because of the smell of the chemicals they use for cleaning” (P3).*

*“I have no appetite for food, especially when he is seriously sick” (P14).*

*“Yes, I experience nausea most of the time because of the hospital smell, besides, I must clean her up and empty her vomitus. I also wash her dirty clothes which are soiled with urine. Madam, I see so many disgusting things in the hospital” (*P1).

*“The hospital scent makes me feel like vomiting I mostly have saliva in my mouth” (*P6)

The consequences of not eating well were evident in their physiological functioning as most of the participants recounted that constipation was one of their major physiological changes. Almost all the participants who reported constipation associated it with inadequate food intake and lack of decent toilets, which necessitate that they practice deliberate bowel control by suppressing the urge to use the toilets.

*“I mostly get constipation. My inability to eat and not to get a good place of convenience makes it difficult to visit the toilet like I usually do” (P1).*

*“Because I cannot eat as I used to, I cannot visit the toilet as I used to” (PI6).*

Fatigue was an experience shared by most family caregivers. Participants recounted getting tired from carrying out care activities, including feeding, bathing, and grooming, assistance with patient ambulation, communication, and handwashing of clothing as part of the caregiving process.

*“Yes, I am human, and running errands daily makes me tired. Sometimes, walking to and from the pharmacy is very stressful*, even in the hospital” (P11).

*“It is not easy at all moving up and down the hospital; at the end of the day, I feel so tired with body pains all over. Sometimes I even have to carry her on my back” (*P12).

Participants reported that sleep disturbance was another physiological change that affected their physical wellbeing. According to them, keeping vigilant over the sick during night hours was one of caregiving’s most challenging and daunting tasks. In this study, it was revealed that the participants stayed awake to keep watch over their sick relatives during critical times.

*“If I say I’ve slept for 2 hours, it’s a lie because of his frequent visits to the toilet at night. Sometimes he would get up, and other times I will have to take him there” (P4).*

Others reported sleep interruptions due to mosquito bites when they slept in open places at the hospital. Some caregivers associated their sleeplessness with excessive thoughts, anxieties, and worries about the poor health status of their family members.

*“In the evening, when I visit him and spend the night at the hospital, since I am sleeping outside there are a lot of mosquitoes so I am unable to sleep but when I get home I can sleep for a while” (P8).*

*“I think a lot these days, so I cannot sleep soundly. My wife is not the same anymore; her abdomen is swollen, she is growing lean, and there is no improvement in her condition” (P9).*

Bodily aches and pains were also some frequent occurrences reported in caregiving roles. They ascribed these aches and pains which they experienced to the lifting and carrying of their sick relatives. Most of the participants practiced self-medication to manage their aches and pains.

*“My son is mostly weak, so I assist him with almost everything, bathing, washing clothes, and supporting him to walk around, and now, I am sick. Currently, I have body pains all over” (P7).*

*“I mostly get body pains. She can’t get up by herself, so I assist her and help her eat by serving her while she feeds herself. Regarding bathing, I take the water to the bathroom and assist her to the bathroom to bathe. I also wash her clothes every day” (P17).*

*“I mostly have headaches and body pains, so I visit the nearby drugstore to purchase some pain relievers” (P8).*

### Social wellbeing of family caregivers

Caregiving poses a challenge to the social wellbeing of family caregivers. The family caregivers reported isolation, role conflicts, financial burden, and employment challenges. These were identified under four subthemes: role strain, social isolation, economic impact and support, and affection/sexual function.

### Role strain

Most family caregivers admitted that caring for a sick relative made it difficult to perform other socially assigned roles, including parenting, marriage, and occupation. Family caregivers who had children could not give their children the needed attention.

*“Everything is a mess; if my husband and eldest daughter are away, my children walk about without help or knowing what to do. I used to wash their clothes, cook, and many more, but now I do not have time”* (P7).

*“I do my best not to neglect my other son, but when my daughter is admitted, and my husband has traveled, I send him to my sister” (*P12*).*

The caregivers who were married and cared for other sick relatives also reported some strain in their relationship with their spouses. For example, some of the participants spent several days out of their homes during the hospitalization of their sick relatives.

*“My wife is in the North and came down recently for a visit, but I couldn’t make time for her, and she was not pleased with that. I pleaded with her to give me some time to care for my brother. She agreed and went back to the North, but I could sense he was not so happy”* (P16).

Some family caregivers had to find alternative arrangements to delegate their usual responsibilities in their businesses and family matters to others so they could be able to give care to their sick family members.

*“For some time now, I have had to entrust my business to someone I can trust to be with my wife to care for her*” (P18).

*“Any time I have to come with him to the hospital, I ask permission from my headmaster, or I swap my teaching period with another teacher” (P3).*

### Social isolation

Caregiving restricts family caregivers from enjoying their everyday social life. Usually, families’ daily social activities include visiting families and friends, receiving visitors, and attending social festive events and ceremonies. Several participants indicated their inability to see others or receive visitors because of lack of time.

*“I do not visit anyone. Someone told me he had stopped visiting me because of my sick daughter” (P13).*

Family caregivers revealed that they spent most of their time attending to their sick family members, depriving them of any leisure time to engage in and enjoy social activities.

*“Whenever he is sick, there are social programs like funerals and weddings; I cannot attend since I always must be with him” (P6).*

Some family caregivers reported needing help to keep up with social events or even following up on local news in the media. Some said their fears of becoming lonely even amid people.

*“I feel lonely because I have no one to talk to, and I am always here alone in the hospital, taking care of my sister” (P17).*

*“Sometimes my heart feels so heavy that I wish I could speak to someone or any of my friends, but the thought of what will follow next, the stigma, stops me” (P1).*

### Financial impact and support

Most family caregivers disclosed they had some form of financial challenges. The reasons for these challenges included the inability to go to work to make money, the cost of care outweighing their income, and the lack of financial support from significant others. Although some family caregivers admitted receiving support from significant others, this support was insufficient to relieve them of the financial burden. Most participants indicated that they had to stop working for some time to care for their sick relatives, which increased their financial burden.

*“Being here to care for my wife has affected my work. I cannot go to work; even when customers call, I have to direct them to other shops. All the money I had on me is finished, and she is not better” (P10).*

*“I don’t even think about my work because I know everything will be fine if my brother gets better. Even if we work or not, only God takes care of us” (P5).*

Family caregivers who were employees revealed they had to ask for permission from their employers or swap duties as ways of alternative work arrangements. Other participants took casual leave from work to care for their sick relatives. They reported that their absence from work had severe financial implications, as some reported pay cuts.

*“Financially, it has not been easy, the company my brother works with pays all his medical bills, but the issue is that I cannot work and make money for myself. I work on a contract basis, but now I cannot go to work. Financially I am hard-pressed*” *(P16).*

The cost of medical care for Hepatitis B was expensive, and this led to financial difficulties. The caregivers indicated that the national health insurance scheme did not cover most drugs prescribed. The patients and families bear the cost of treatment alone with no support from the healthcare system.

*“Every month we visit the hospital to purchase some medications prescribed by the doctor and go for some laboratory test which includes liver test. Madam, it hasn’t been easy in terms of purchasing the drugs” (P2).*

*“It has been stressful in terms of finances. From buying medication, paying for laboratory investigation, and transport fares* “*(P3).*

*“There is no help from anywhere. I have four sons, all depending on me, and now even the little we have to use for food has to be saved to buy drugs. It has not been easy, and currently, there has been an increment in the price of the medications. My husband has no better support, so I am facing financial difficulty” (P2).*

Participants indicated financial support and support with the physical care of the care recipients as very necessary. Participants reported receiving financial aid and help with physical care from their family and other sources, which have been very helpful. A few participants admitted that support in diverse forms from other people was essential for their social wellbeing.

*“My mother is helping to put things in order. I have two other children who are also working and earning something to support my mother, wife, and employed children are all helping, so in terms of financing, we are a little stable” (P11).*

*“My husband’s family members are all doing their best by contributing some money to help us care for him. Now the family’s money is finished. It’s up to me when he needs anything; only God can help me” (P4)*.

***“****One of our daughters helps with bathing, feeding, and other things. It has helped me”* (P9)

However, most participants reported a lack of support to provide physical care and inadequate financial support.

*“Now I am in a difficult situation with someone to help. Because I am caring for her, I cannot go to work. Her father is far away, but her mother is not on good terms with us, so since her daughter got sick, she has never visited her before” (P10).*

*“Financially, it has not been easy, we are both mechanics, and now that he is sick, everything is on me. Once in a while, some friends and family members bring something small, but it just does not get anywhere” (P15).*

### Affection/sexual function

Family caregivers asserted they experience challenges meeting their affection and sexual functioning needs. All the participants but one was married, and they admitted that mostly, their sexual needs were unmet. For example, family caregivers caring for their spouses reported they had to suppress their sexual desires since their spouses were consciously sick. When the caregiver was providing care to another family member, the caregiver, and spouse would be deprived of affection as one spends time in the hospital.

*“Madam, my appetite for sex has gone on an extended holiday (smiles). I want him to conserve his energy, so I don’t worry him about sex like I used to do. I only make demands occasionally when I see he is better” (P1).*

*“It has affected our sex life as a couple. Seeing her alone makes me sad I want her to get well, so sex is not on my mind now” (P9).*

Some participants who provided care to family members other than their spouses reported that their spouses were equally worried, so they did not burden them with sexual demands. Others did not have the support of their spouses and hence reported a strain in their relationship with their spouses.

*“Madam, when there is happiness, you can have time for pleasure as a couple. Currently, the thought of fun is not in our minds because anytime we go to bed*, *the only thing we do is think about the wellbeing of our son” (P7).*

*“I spend the night at the hospital, return home in the morning, and return to the hospital in the afternoon. It has affected our sex life, but we understand each other because of the current situation with my uncle. It has not affected us” (P8).*

*“Since my daughter was diagnosed with this illness, my husband has changed. I think it is because the girl is not his real daughter. He makes sexual demands, not caring whether I am tired. Sometimes when my daughter is very ill, and I want to sleep beside her at night, he will refuse” (P13).*

*“Sometimes my daughter was seriously sick, and he was making sexual advances. I told him I was not in the mood because I worried about my daughter. He got upset with me” (P12).*

## Discussion

This study explored the physical and social wellbeing of family caregivers of persons with hepatitis B-associated chronic liver disease. Our findings have shown that family caregivers encounter physical and social challenges while providing care due to the demanding and prolonged nature of the disease. The physical difficulties included physical and physiological changes, mainly due to the demanding nature of caregiving. The nature of caregiving makes family caregivers focus on the caregiving task to the detriment of their physical wellbeing. Regarding social challenges, role strain, social isolation, financial impact, affection/sexual function, and support were the areas of concern to the participants. Family caregivers spend a significant amount of their time providing care. They had little or no time for leisure and other family roles. They suffered financial challenges since they had little or no time to work. Also, the lack of financial support worsened their financial burden.

Physical and physiological body changes that family caregivers experienced, which affected their physical wellbeing, were stated as; weight loss, inadequate personal grooming and change in physique. Fatigue, sleeplessness, loss of appetite, aches/pains, and constipation were also reported. The physical effect of caregiving can manifest in symptoms of sleep disturbance, gastrointestinal disorders, fatigue, loss of strength, and weight loss [22]. Northouse et al. similarly said that caregiving has a bearing on family caregivers and can adversely affect the caregivers’ health outcomes [23]. The stress associated with caregiving is often evident in the decline of the family caregiver’s health status. After taking up the caregiver role, caregivers’ health significantly worsened.

Concerning physical changes, deterioration in physical health was shown in this study partly due to physical exertions in caring for the family member, less time available for exercise, and preparing healthy meals. Burton et al. identified that many caregivers reported concerns about a burden on their time as they must adjust their schedules and relinquish valued personal activity. The family caregiver’s primary focus is the sick family member. Caregivers usually have inadequate time to cater to their unique needs, which invariably causes a decline in physical health [24]. They plan their lives according to the patient’s schedules and conditions and do not have enough time to care for their needs [24]. This suggests that family caregivers should be encouraged to be attentive to changes in their physical wellbeing and seek appropriate assistance. This will help caregivers continue providing care without compromising their health [23].

Regarding physiological change**s**, the research finding further identified that family caregivers suffered fatigue from assisting the care recipient in performing various tasks such as feeding, personal hygiene needs, and ambulation. This finding is congruent with Nielsen et al. [25] that the prolonged nature and extensive tasks the caregiver performs can cause caregiver fatigue. The physically demanding care tasks leave the family caregivers exhausted and affect their quality of life in physical wellbeing [26]. Family caregivers are said to suffer from tiredness, anger, depression, a sense of helplessness, guilt, isolation, loss of freedom, fear, vulnerability, and neglect of their health. It is suggested that other family members should be encouraged to share caregiving tasks and to support the family caregiver to help prevent fatigue.

It was revealed in this study that family caregivers usually experienced aches/pains with the magnitude of caregiving responsibilities. Wittenberg et al. [27] report similar findings that the obstacles influencing family caregivers’ physical wellbeing include; aches and pains from providing physical to patients. Another study also said that caregivers lacked the requisite knowledge in applying body mechanics and lifting techniques while providing care, which inadvertently resulted in body pains[28]. Family caregivers should be educated on the use of body mechanics and appropriate lifting techniques to help prevent or reduce body pains.

Sleep disturbance was another experience revealed in this study that affects the physical wellbeing of family caregivers. Family caregivers encountered disruptions in sleep in the caregiving trajectory. This finding is consistent with similar results, which reported sleep disturbance as one of the problems associated with family caregiving [29]. Being constantly awake made family caregivers susceptible to tiredness and other physical challenges. Also, family caregivers experienced sleep disturbances as they mostly kept awake worrying about the state of health of the care recipient. Though providing care for a family member meant inadequate time for the caregiver’s upkeep, the lack of a suitable place to sleep compounds the problem of sleeplessness. It is worth stating that support from significant others to family caregivers in performing caregiving tasks is vital to improving their sleep quality and physical wellbeing.

Another finding from this study revealed that family caregivers experience a loss of appetite in the care trajectory. For example, the hospital environment and its associated odor caused loss of appetite in some participants, which inadvertently resulted in weight loss [22]. The physical stress associated with caregiving also resulted in weight loss in most participants. Other participants out of worry about their relative’s illness, could not eat. This mostly led to constipation, as revealed in this study [22]. Support for family caregivers in the form of practical assistance from both formal and informal sources would help alleviate the challenges that come with caregiving [22, 30].

Isolation, role adjustment, financial stress, occupation problems, leisure, and affection/sexual function were all highlighted by participants as they discussed their social wellbeing. These were grouped mainly under; role strain, social isolation, financial impact, affection/sexual function, and support. Regarding role strain, the study’s findings revealed that most family caregivers struggled to combine caregiving with other roles and responsibilities. Again, caregiving competes with leisure time, usually spent with family members to help sustain healthy family relations [31]. For example, caregiving made it difficult for family caregivers who were parents to adequately attend to the needs of their children [32]. Some family members often felt stuck in the caregiver role, resulting in conflicts with their personal and work lives. The time required for caregiving deprived caregivers of time to care for other family members [32]. It is important that family caregivers make conscious efforts to seek help from other family members periodically. This would give the caregivers time to cater to other responsibilities.

The study further revealed that family caregivers could not enjoy their social life and felt socially isolated. Similar findings have indicated that family caregivers caring for sick relatives and other family members have limited time and have truncated social networks to be able to provide care [33]. Family caregivers stand a higher risk of experiencing social isolation [34]. Family caregivers had limited social engagements because they spent most of their time on caregiving tasks, negatively impacting their social wellbeing [35]. This suggests that it is essential to create social support groups and encourage family caregivers to join these groups to enable them to have access to resources aimed at reducing the feeling of isolation and enhancing their social wellbeing [36].

Regarding financial impact, the study also revealed that almost all family caregivers experienced economic challenges of varying degrees. Participants indicated that the cost of treatment posed a tremendous financial burden. Similar findings have emerged from other studies that financial burden and insufficient resources are the main factors associated with caregiver burden [37, 38]. Sarris et al. [24] also identified that economic challenges are the worst aspect of family caregivers’ experience. Caregiving impacts the caregiver’s employment which subsequently affects their income. As revealed in this study and some studies elsewhere, most family caregivers had to give up or reschedule their work to be able to provide care for their sick relatives [39]. Some family caregivers forfeit employment and their career indefinitely to provide care [24, 40]. Most employed family caregivers experienced difficulty managing their paid work and caregiving responsibilities. Similarly, Gardiner et al. [41]reported that the financial costs of caregiving were substantial. This suggests that family caregivers should be aware of the direct and indirect costs involved in treating their sick relatives. This will help them prepare for the financial challenges ahead [42]. The high cost of care stated previously reiterates the need for financial support and adequate insurance coverage. Also, workplace policy reforms on reducing salaries with absence from work should exempt employed family caregivers [24].

The study further identified that the affection/sexual function aspect of the family caregiver’s life is affected while providing care. Family caregivers who doubled as spouses had conflicts with their spouses due to neglect of the needs of the spouses. As reported in this study, family caregivers who cared for their spouses had to consciously suppress their sexual feelings out of compassion for their spouses. On the other hand, a few caregivers indicated improved relations with a sick family member since the caregiving made them spend most of their time together. Family caregivers stated varied reasons for these changes in affection/sexual function. Bauer [31] confirms that a longer care duration can result in marital dissatisfaction. Some family caregivers experienced decreased libido associated with sadness and anxiety. Other family caregivers had a decreased libido due to the physical changes in their spouse resulting from illness and treatment. The impaired relations between spouses and other close ties can also be related to fear of infection transmission. For example, family caregivers caring for their spouses hesitated to have intimate sexual relations, as revealed in this study [43]. This implies that much more education is needed especially on the transmission of hepatitis B to help reduce fear and stigmatization [11, 44].

The findings from this study identified support as an essential component in promoting the social wellbeing of family caregivers. The study revealed that most family caregivers desired support in several ways while providing care that was lacking. However, some caregivers in this study reported receiving financial support from other family members, Non-Governmental Organizations, and friends to help cater to the cost of providing care [45]. In a related study, family caregivers repeatedly requested a resource they could use without the patient, specifically tailored to their needs as caregivers [46]. However, most healthcare delivery policies focus mainly on the sick individual with little or no support for family caregivers [47]. Empirical data suggests that having more formal social support, such as nursing homes, transportation, support groups, and financial aid, can help patients and caregivers cope with their responsibilities[48]. It was revealed that support from significant others to family caregivers in providing physical care is necessary to ease the challenges associated with caregiving. Some male participants who were caring for their spouses sometimes enjoyed support from other female relatives in the area of physical care. This corroborates with Bertogg & Strauss, who identified that husbands are likelier to share caregiving with an informal helper, while wives are potential sole caregivers [49]. This suggests more robust advocacy for policies that will aim at reducing the cost of treatment and a total insurance cover for the prevention and treatment of hepatitis B infection. Also, the provision of informal care should be a shared responsibility for all family members to reduce the burden on family caregivers.

### Strengths and limitations of the study

This study is among the few on family caregivers of persons with HB-associated chronic liver disease in Africa. A limitation of the study was the lack of opportunities to provide some basic interventions such as education on relaxation techniques and patient efficient management strategies which can reduce family caregiver’s stress. Future studies should consider interventions to support the family caregivers.

## Conclusions

Caregiving puts a toll on the physical and social wellbeing of family caregivers. This study has revealed the challenges family caregivers of persons with hepatitis B-associated chronic liver disease encounter concerning their physical and social wellbeing in the caregiving trajectory. Most family caregivers combine caregiving with other family and social responsibilities. They suffer physical and physiological changes as they neglect their wellbeing. Family caregivers feel isolated and encounter financial difficulties. They need more support from other family members and organizations.

## Data Availability

The datasets generated during and/or analysed during the current study are available from the corresponding author on reasonable request.

## Abbreviations

CCTH: Cape Coast Teaching Hospital
HBV: Hepatitis B Virus
OPD: Out-patient Department
